# Migrant’s access to preventive health services in five EU countries

**DOI:** 10.1186/s12913-017-2549-9

**Published:** 2017-08-23

**Authors:** Aldo Rosano, Marie Dauvrin, Sandra C. Buttigieg, Elena Ronda, Jean Tafforeau, Sonia Dias

**Affiliations:** 1 Roman Academy of Public Health, Rome, Italy; 2 Department of Social Inclusion, National Institute for Public Policies Analysis, INAPP, Rome, Italy; 3 Institute of Health and Society, Université catholique de Louvain, Louvain, Belgium; 4 Department of Health Services Management, Faculty of Health Sciences, University of Malta, Msida, Malta; 5 University of Alicante, Alicante, Spain; 6 Scientific Institute of Public Health, Brussels, Belgium; 7 Global Health and Tropical Medicine, GHTM, Instituto de Higiene e Medicina Tropical, IHMT, Universidade Nova de Lisboa, Rua da Junqueira, 100, 1349-008 Lisbon, Portugal

**Keywords:** Preventive health services, Screening, Migrant health, Multilevel analysis, National survey

## Abstract

**Background:**

Preventive health services (PHSs) form part of primary healthcare with the aim of screening to prevent disease. Migrants show significant differences in lifestyle, health beliefs and risk factors compared with the native populations. This can have a significant impact on migrants’ access to health systems and participation in prevention programmes. Even in countries with widely accessible healthcare systems, migrants’ access to PHSs may be difficult. The aim of the study was to compare access to preventive health services between migrants and native populations in five European Union (EU) countries.

**Methods:**

Information from Health Interview Surveys of Belgium, Italy, Malta, Portugal and Spain were used to analyse access to mammography, Pap smear tests, colorectal cancer screening and flu vaccination among migrants. The comparative risk of not accessing PHSs was calculated using a mixed-effects multilevel model, adjusting for potential confounding factors (sex, education and the presence of disability). Migrant status was defined according to citizenship, with a distinction made between EU and non-EU countries.

**Results:**

Migrants, in particular those from non-EU countries, were found to have poorer access to PHSs. The overall risk of not reporting a screening test or a flu vaccination ranged from a minimum of 1.8 times (colorectal cancer screening), to a high of 4.4 times (flu vaccination) for migrants. The comparison among the five EU countries included in the study showed similarities, with particularly limited access recorded in Italy and in Belgium for non-EU migrants.

**Conclusions:**

The findings of this study are in accordance with evidence from the scientific literature. Poor organization of health services, in Italy, and lack of targeted health policies in Belgium may explain these findings. PHSs should be responsive to patient diversity, probably more so than other health services. There is a need for diversity-oriented, migrant-sensitive prevention. Policies oriented to removing impediments to migrants’ access to preventive interventions are crucial, to encourage more positive action for those facing the risk of intersectional discrimination.

## Background

Preventive health services (PHSs) form part of primary healthcare with the aim of screening for and preventing diseases. The goal of a PHS is to reduce morbidity and mortality through prevention and early detection of preventable diseases [1]. However, barriers to access to preventive health services can be higher than to other health services; this may even be more problematic for some vulnerable groups such as migrants [2]. Migrants show significant differences in lifestyle, health beliefs and risk factors compared with the national populations that may prevent them from accessing health services and participating in prevention programs [3]. Even in countries with widely accessible healthcare systems, migrants’ access to PHSs may be problematic.

Migrants encounter legal, financial, cultural and geographical barriers to health care access including absence of health insurance coverage, linguistic and cultural problems, and lack of access to information (affecting the poorly educated and those with a poor health literacy). Other factors overlie all of these barriers, such as the inability to afford the direct financial costs of care (affecting low-income groups), lack of mobility (affecting disabled and elderly persons), as well as gender issues [4]. As a consequence, being a migrant may include any of these barriers.

In this study, we analysed the following PHSs: screening for breast cancer, cervical cancer and colorectal cancer and flu vaccination. The choice was conditional on the availability of relevant information in the selected data sources, the national Health Interview Surveys (NHIS).

Secondary cancer prevention programs are particularly important for migrants because they often underuse preventive care, fail to attend follow-up consultations, and lack of cancer awareness. Migrants are more likely to receive a diagnosis of cancer at an advanced stage [5]. Limited access to screenings is one possible reason for this. This problem tends to decrease as the length of stay of migrants in the host country increases [2].

Breast cancer is the most commonly diagnosed cancer among women worldwide [6]. Breast cancer screening is performed through mammography and breast self-examination and should be started between the ages of 40 and 50 (or earlier depending on family history). Among migrants living in the United States of America (USA), large disparities exist for secondary prevention, as migrants tend to adopt a rather passive attitude toward screening [2].

Cervical cancer is the second most common cancer in women from developing countries [6]. In many countries, screening uptake in migrant women is far below target and significantly lower than for the national population. Reasons for non-participation in cervical cancer screening are mostly related to language problems and dissatisfaction with health care professionals. Religion may also play an important role, women may be reluctant to be examined by male doctors [2].

Colorectal cancer is much more common in developed countries and is associated with dietary habits and environmental risk factors. It is recommended that screening starts at around the age of 50 using faecal occult blood tests and endoscopic exams. Colorectal cancer is the second most common cancer in Europe, causing over 200,000 deaths per year [7]. Population-based colorectal cancer screening has proven to be effective in reducing colorectal cancer incidence and mortality. [8] In the USA, colorectal screening tends to be lower for migrants than for the national American population. However, screening patterns converge towards that of the national population as the length of stay in the country increases [2].

Influenza is a vaccine-preventable disease that infects around 15% of European population each year. Older people, young children and those with chronic conditions suffer the most although everyone is at risk of developing serious complications that may result in death [9]. In the USA, influenza vaccination coverage among most racial/ethnic groups has increased in recent years, but substantial racial and ethnic disparities remain in most age groups [10]. Evidence from European countries is limited to a few countries [11, 12], with migrants having a lower probability of receiving the influenza vaccine compared with national population.

As highlighted above, studies on the access of migrants to PHSs are more common in the USA. Conversely, few studies in Europe have investigated migrants’ access to PHSs. The available evidence shows that European Union (EU) countries’ migrants have lower rates of preventive service utilization than national populations, despite the narrowing gap in recent years for some of these services, such as breast and cervical cancer screening [13, 14]. According to the European Charter of Fundamental Rights [15] “*Everyone has the right of access to preventive health care*” - but - “*under the conditions established by national laws and practices”*. Meeting the health needs of migrants depends on the health system of the host country. Despite the EU’s declared intention to harmonize their entitlements, there are still significant differences also for documented migrants. Although the law may grant migrants certain entitlements to healthcare coverage, administrative procedures or cultural barriers often prevent them from exercising these rights [16].

The data available from some EU countries suggest the presence of inequalities in health care use, since migrants tends to have a greater reliance on emergency services, because of inadequate access to other services, such as primary and/or specialist care [17]. Particularly worrisome are the low utilization rates of antenatal and paediatric care, and preventive services [18]. High quality studies based on comparable data and adjusting for selected factors are needed to make valid decisions on how to secure equity in the access of migrant to health services. [17]. This study aimed at comparing access to preventive health services between migrants and national populations, while considering some factors that may confound or interact with the relationship between access to PHSs and migrant status.

## Methods

This study was a retrospective period prevalence study, based on secondary data from the national Health Interview Surveys (HISs) of Belgium, Italy, Malta, Portugal and Spain. Data were respectively collected in 2008 for Malta, 2011 for Spain, 2012–13 for Italy, 2013 for Belgium and 2014 for Portugal. The National NHIS are cross-sectional survey intended to provide nationally representative estimates on a wide range of health status and utilization measures among the noninstitutionalized population. They are conducted in many European countries with different periodicity with a large sample size and allow to provide reliable statistics on the principal health domains. Data from NHIS are currently used to calculate various European Core Health Indicators (ECHI) consisting in a comparable health information and knowledge system for monitoring health at EU level [19].

### Data source and variables of interest

We collected individual information from HISs regarding access to mammography, Pap smear tests, colorectal cancer screening and flu vaccination.

Four indicators of PHS access were considered, according to the European Core Health Indicators’ definitions [20]:

(1) Percentage of women (aged 50–69) reporting a mammography in the past 2 years;

(2) Percentage of women (aged 20–69) reporting a Pap smear test in the past 3 years;

(3) Percentage of persons (aged 50–74) reporting a colorectal cancer screening in the past 2 years;

(4) Percentage of persons aged 65 and over reporting a vaccination against flu in the past 12 months.

Appendix 1 presents additional information about the availability of other items on screening programs in national HIS and the existence of a screening program.

Demographic characteristics retrieved from the HISs were citizenship, age and sex of participants. Migrant status was defined according to citizenship as the migrants included in the national HIS are migrants with a legal permit of residence in the host country. Migrants were distinguished between migrants originating from a European country member of the EU and migrants of non-European countries.

Additionally, two variables that may influence the relationship between migrant status and access to PHSs were retrieved from the HISs database: level of education and the presence of disability. Education was classified into three levels: no education/primary, secondary and higher education. The presence of disability was coded as a dichotomous variable according to the reported long-term limitation, irrespective of its severity.

### Analysis

Analysis was computed for nationals, migrants with European citizenship (EU) and migrants with a non-EU citizenship. In order to compare the percentage of migrants accessing PHSs with that of nationals, odds ratios (ORs) were estimated using logistic regression models adjusted for potential confounders (sex, presence of disability, education), using nationals as a reference. Country-specific ORs were calculated, as well as overall risks.

To consider the country level factors, a mixed-effects multilevel model was used to estimate the overall ORs. The multilevel regression considered that the individual probability was also statistically dependent on the country of residence [21]. The Intraclass Correlation Coefficient (ICC) was used to test the need to use multilevel models. The ICC varies from +1 when group risks differed, but within any group there is no variation, to −1/(n-1) when group means are equal but the within-group variation is large [22]. Random effects parameters were used to explain the correlation and variation of the latent mean values around the population average values. The ICC was large enough to account for the clustering (country) effect when analysing mammography (ICC = 0.15; 95% C.I.: 0.05–0.39) and colorectal cancer screening (ICC = 0.19; 95% C.I.: 0.06–0.46). Conversely the ICC was only slightly significant when analysing Pap smear testing (ICC = 0.01; 95% C.I.: 0.00–0.34) and vaccination against flu (ICC = 0.04; 95% C.I.: 0.01–0.13). Therefore, multilevel models were only used for analysing mammography and colorectal cancer screening whereas one-level logistic regression models were used for analysing Pap smear testing and flu vaccination, since the observations within clusters (countries) are no more similar than observations from different clusters.

Based on the HIS surveys, we also calculated the percentage of women invited by national or regional screening programmes, by citizenship, to assess the capacity of screening programmes to attract migrant women. The STATA software, release 13, was used to analyse the data [23].

## Results

### Participants

Table 1 presents the sociodemographic characteristics of the subjects included in the analysis. A total of 151,311 subjects were analysed: 5480 from Belgium, 102,953 from Italy, 3668 from Malta, 18,203 from Portugal and 21,007 from Spain. The sample included 46% of men, 54% of women, 6% of migrants and 94% of nationals.

**Table 1:** Total number of interviewed subjects (N), percentage of males and percentage of migrants by age class and country

	Countries															Total		
	Belgium (*n* = 5480)			Italy (*n* = 102,953)			Malta (*n* = 3668)			Portugal (*n* = 18,209)			Spain (*n* = 21,007)					
	N	% males	% migrants	N	% males	% migrants	N	% males	% migrants	N	% males	% migrants	N	% males	% migrants	N	% males	% migrants
Participants																		
Age class																		
15–19	0			5771	52.1%	6.6%	284	47.5%	0.7%	724	50.7%	2.1%	740	53.5%	10.5%	7519	51.9%	6.3%
20–24	322	0.0%	17.1%	5974	50.9%	7.4%	293	50.5%	1.0%	694	46.8%	3.5%	916	48.7%	14.1%	8199	48.3%	8.0%
25–29	353	0.0%	27.8%	5761	51.4%	11.0%	279	47.3%	1.4%	715	44.6%	4.3%	1126	49.8%	16.7%	8234	48.2%	11.6%
30–34	390	0.0%	24.9%	6576	48.6%	12.2%	277	42.6%	4.0%	1128	44.3%	4.3%	1631	47.9%	16.2%	10,002	45.9%	12.3%
35–39	370	0.0%	19.7%	8144	48.8%	10.9%	240	43.8%	2.1%	1517	47.9%	3.2%	2010	51.4%	13.4%	12,281	47.5%	10.4%
40–44	389	0.0%	21.1%	9320	49.1%	8.2%	267	47.2%	2.6%	1671	45.8%	3.7%	1941	51.1%	10.5%	13,588	47.5%	8.2%
45–49	429	0.0%	14.5%	9741	48.4%	6.0%	338	48.5%	4.4%	1480	46.4%	2.2%	1881	48.0%	9.3%	13,869	46.6%	6.2%
50–54	747	49.4%	14.9%	8847	48.5%	5.2%	354	46.6%	2.3%	1542	45.8%	2.9%	1693	50.2%	6.7%	13,183	48.4%	5.6%
55–59	767	49.2%	9.9%	7919	49.5%	4.0%	324	48.5%	2.2%	1473	45.1%	2.0%	1619	46.1%	5.4%	12,102	48.5%	4.2%
60–64	683	49.2%	8.5%	7897	47.6%	2.0%	338	45.6%	2.7%	1558	42.9%	1.5%	1554	46.3%	3.9%	12,030	46.8%	2.6%
65–69	596	48.8%	9.2%	7107	47.1%	1.1%	181	47.5%	3.9%	1533	42.7%	1.2%	1480	44.3%	2.1%	10,897	46.2%	1.7%
70–74	434	44.7%	7.6%	6695	46.3%	0.8%	203	45.3%	1.5%	1319	38.9%	1.4%	1251	38.9%	2.4%	9902	44.3%	1.4%
75–79				5469	44.3%	0.6%	153	39.2%	2.0%	1259	37.7%	0.9%	1327	35.4%	1.4%	8208	41.8%	0.8%
80–84				4209	39.2%	0.3%	91	40.7%	3.3%	979	35.8%	0.6%	1023	35.6%	1.9%	6302	38.1%	0.7%
> 84				3523	32.5%	0.5%	46	43.5%	2.2%	611	36.5%	0.5%	815	30.2%	0.7%	4995	32.7%	0.6%
Total	5480	28.6%	14.6%	102,953	47.7%	5.5%	3668	46.3%	2.4%	18,203	43.6%	2.3%	21,007	45.9%	8.0%	151,311	46.2%	5.7%

### Access to PHS

Tables 2, 3, 4 and 5 presents the percentage of subjects reporting a screening test or a flu vaccination according to the type of test and the country of residence. Excluding Malta, in all countries, access to Pap smear tests, colorectal cancer screening and flu vaccination was lower for migrants from non-EU countries than for nationals and EU migrants. Access to mammography was therefore the lowest for those from EU countries when compared to non EU migrants and nationals.

**Table 2:** Proportion of Pap smear test in the past 3 years (*n* = 17,724)

	Citizenship						Total
	Nationals		EU migrants		Non- EU migrants		
	N (%)	CI 95%	N (%)	CI 95%	N (%)	CI 95%	
Country							
Belgium	677 (70.4)	67.5–73.3	63 (69.9)	60.4–79.3	51 (46.9)	37.5–56.3	69.5
Italy	9984 (66.8)	66.1–67.6	352 (55.7)	51.8–59.5	727 (48.5)	46.0–51.1	65.8
Malta	585 (57.9)	54.8–60.9	7 (70.8)	42.5–99.2	8 (55.6)	29.9–81.2	58.1
Portugal	2593 (63.2)	61.7–64.7	39 (50.6)	39.5–61.8	46 (63.8)	52.7–74.9	63.1
Spain	2287 (68.5)	66.9–70.1	97 (58.2)	50.7–65.7	208 (63.1)	57.9–68.3	67.8
Total	16,126 (66.5)	65.9–67.1	558 (58.3)	55.2–61.4	1040 (53.1)	50.9–55.3	65.7

Data were collected for women aged 20–69

**Table 3:** Proportion of colorectal cancer screening in the past 2 years (*n* = 49,259)

	Citizenship						Total
	Nationals		EU migrants		Non- EU migrants		
	N (%)	CI 95%	N (%)	CI 95%	N (%)	CI 95%	
Country							
Belgium	1789 (16.5)	15.8–17.2	115 (20.7)	17.3–24.1	35 (7.9)	5.4–10.4	16.6
Italy	32,702 (12.6)	12.4–12.7	313 (7.9)	7.1–8.8	683 (6.3)	5.9–6.8	12.4
Malta	1264 (2.2)	2.12–2.4	28 (12.5)	8.17–16.8	1 (50.0)	0.0–100.0	2.6
Portugal	5258 (27.4)	26.7–28.0	70 (17.6)	13.9–21.4	39 (18.8)	13.4–24.1	27.2
Spain	6667 (6.3)	6.2–6.5	135 (2.9)	2.4–3.4	160 (8.6)	7.3–9.8	6.3
Total	47,680 (13.6)	13.5–13.7	661 (10.8)	10–11.6	918 (7.5)	7.0–7.9	13.5

Da**t**a are collected for persons aged 50–74

**Table 4:** Proportion of flu vaccination in the past 12 months (*n* = 34,435)

	Citizenship						Total
	Nationals		EU migrants		Non- EU migrants		
	N (%)	CI 95%	N (%)	CI 95%	N (%)	CI 95%	%
Country							
Belgium	457 (51.4)	48.1–54.7	43 (35.8)	27.2–44.4	10 (50.0)	28.1–71.9	50.4
Italy	26,568 (36.0)	35.7–36.4	352 (11.8)	10.6–12.9	1074 (10.1)	9.5–10.6	35.1
Malta	275 (56.6)	52.2–61.0	6 (64.7)	33.9–95.5	0		56.8
Portugal	3139 (44.1)	42.9–45.2	40 (23.1)	16.8–29.3	4 (100.0)	100.0–100.0	43.9
Spain	2410 (58.4)	56.9–59.9	30 (46.4)	34.3–58.6	27 (43.8)	31.4–56.1	58.2
Total	32,849 (39.7)	39.4–40.1	471 (20.3)	18.7–21.9	1115 (11.9)	11.3–12.6	38.9

Data are collected for persons aged 65 and over

**Table 5:** Proportion of mammography attendance in the past 2 years (*n* = 7837)

	Citizenship						Total
	Nationals		EU migrants		Non- EU migrants		
	N (%)	CI 95%	N (%)	CI 95%	N (%)	CI 95%	
Country							
Belgium	268 (73.4)	68.8–77.9	16 (73.3)	54.8–91.9	5 (66.7)	32.9–100	73.3
Italy	5402 (63.7)	62.7–64.7	127 (38.0)	32.8–43.3	202 (43.9)	39.4–48.4	62.9
Malta	404 (30.9)	28.4–33.4	5 (61.5)	28.1–95.0			31.6
Portugal	641 (80.9)	78.1–83.6	17 (52.8)	35.5–70.0	10 (61.5)	37.9–85.2	80.4
Spain	668 (79.1)	76.3–81.8	41 (45.3)	35.1–55.6	31 (65.6)	52.0–79.1	77.9
Total	7383 (67.9)	67.0–68.8	206 (47.0)	42.4–51.7	248 (49.5)	45.1–53.9	67.2

Data are collected for women aged 50–69

Table 6 presents the percentage of women attending organized national or regional screening programmes, estimated from the data of national HISs considered in this study. Self-reported attendance for such screening was the highest in Italy and Spain, and the lowest in Belgium and Malta. The proportion of migrant women attending screening programmes is 46% lower than nationals for breast cancer screening but only 5% lower for Pap smear tests. In Italy and Belgium, this proportion was higher among migrant women than nationals.

**Table 6:** Percentage of women invited by national or regional screening programmes in 5 European countries, by citizenship and by country

	Screening programs			
	Mammography		Pap smear test	
	Nationals (%)	Migrants (%)	Nationals (%)	Migrants (%)
Country				
Belgium	21.9	9.3	3.0	3.7
Italy	37.7	29.6	34.2	37.7
Malta	3.2	0.0	1.1	0.0
Spain	50.8	29.3	18.5	11.6
Total	38.8	26.6	29.4	28.1

### Risks of not reporting access to PHSs

Tables 7, 8, 9 and 10 presents the risks o migrants, in terms of odds ratios, of self-reported lack of access to PHSs as well as the risks associated with selected variables of influence. The overall risk of not reporting PHSs use was the lowest for colorectal cancer screening and the highest for flu vaccination for all migrants. The overall risks of not attending the PHSs were the highest in Italy and lowest in Belgium (excluding Malta), all PHSs compounded.

**Table 7:** Odds Ratio of not reporting a mammography in the past 2 years

	Citizenship					
	Migrants		EU Migrants		Non-EU Migrants	
	OR	CI 95%	OR	CI 95%	OR	CI 95%
Migrant status						
National (ref)						
Migrant status	2.46	2.14–2.89	2.68	2.18–3.29	2.36	1.97–2.82
Educational level						
No education / basic (ref)						
Secondary	0.71	0.66–0.76	0.71	0.66–0.76	0.70	0.66–0.75
High	0.65	0.55–0.71	0.66	0.60–0.73	0.66	0.60–0.73
Countries						
Belgium	0.94	0.54–1.65	0.90	0.48–1.68	1.11	0.33–3.65
Italy	2.61	2.20–3.10	3.16	2.37–4.20	2.35	1.90–2.90
Malta	0.35	0.11–1.11	0.35	0.11–1.11	-	-
Portugal	3.61	2.15–6.09	4.28	2.19–8.38	2.89	1.29–6.45
Spain	3.10	2.25–4.28	4.91	3.08–7.82	2.07	1.33–3.23

Data are collected for women aged from 50 to 69 years

**Table 8:** Odds Ratio of not reporting a Pap smear test in the past 3 years

	Migrants		EU Migrants		Non-EU Migrants	
	OR	CI 95%	OR	CI 95%	OR	CI 95%
Migrant status						
National (ref)						
Migrant status	1.70	1.59–1.82	1.53	1.36–1.71	1.80	1.65–1.96
Educational level						
No education / basic (ref)						
Secondary	0.63	0.60–0.66	0.63	0.60–0.66	0.64	0.61–0.67
Higher	0.55	0.52–0.59	0.56	0.53–0.60	0.57	0.53–0.60
Countries						
Belgium	1.42	1.08–1.86	1.09	0.79–1.52	2.46	1.56–3.88
Italy	1.95	1.79–2.13	1.71	1.48–1.97	2.10	1.89–2.34
Malta	0.83	0.43–1.59	0.58	0.24–1.43	1.29	0.50–3.33
Portugal	1.43	1.07–1.91	2.13	1.36–3.36	1.11	0.77–1.61
Spain	1.40	1.20–1.64	1.73	1.32–2.27	1.28	1.07–1.54

Data are collected for women between 20 and 69 years

**Table 9:** Odds Ratio of not reporting a colorectal cancer screening in the past 2 years

	Migrants		EU Migrants		Non-EU Migrants	
	OR	CI 95%	OR	CI 95%	OR	CI 95%
Migrant status						
National (ref)						
Migrant status	1.57	1.33–1.86	1.20	0.95–1.52	1.97	1.55–2.50
Educational level						
No education / basic (ref)	1.00		1.00		1.00	
Secondary	0.90	0.84–0.95	0.90	0.85–0.96	0.89	0.84–0.95
Higher	0.82	0.76–0.90	0.84	0.77–0.90	0.82	0.76–0.90
Gender						
Men (ref)						
Women	1.11	1.05–1.17	1.11	1.06–1.17	1.11	1.05–1.17
Estimates by country						
Belgium	0.88	0.59–1.32	0.75	0.49–1.15	2.11	0.64–6.95
Italy	1.99	1.56–2.53	1.71	1.15–2.53	2.15	1.59–2.91
Malta	0.14	0.05–0.41	0.18	0.05–0.56	0.02	0.00–0.37
Portugal	1.70	1.09–2.67	1.76	1.00–3.10	1.62	0.78–3.36
Spain	1.13	0.70–1.81	2.49	0.92–6.78	0.76	0.44–1.31

Data are collected for men and women aged between 50 and 74 years

**Table 10:** Odds Ratio of not a reporting a vaccination against flu in the past 12 months

	Migrants		EU Migrants		Non-EU Migrants	
	OR	CI 95%	OR	CI 95%	OR	CI 95%
Migrant status						
National (ref)						
Migrant status	3.69	3.24–4.21	2.85	2.32–3.49	4.42	3.72–5.25
Educational level						
No education / basic (ref)						
Secondary	0.65	0.61–0.69	0.65	0.62–0.69	0.65	0.62–0.69
Higher	0.65	0.61–0.70	0.65	0.61–0.70	0.65	0.61–0.70
Gender						
Men (ref)						
Women	0.88	0.85–0.92	0.89	0.86–0.92	0.89	0.86–0.92
Estimates by country						
Belgium	1.62	1.03–2.55	1.86	1.11–3.13	1.03	0.422.51
Italy	4.63	3.94–5.45	4.18	3.08–5.67	4.82	3.98–5.82
Malta	0.80	0.29–2.21	0.80	0.29–2.21		
Portugal	2.43	1.27–4.65	2.19	1.14–4.24		
Spain	1.56	1.05–2.31	1.45	0.85–2.46	1.71	0.96–3.04

Data are collected for men and women aged of 65 years and over

When comparing EU migrants and non-EU migrants, higher risks were found for non-EU migrants, except in the case of mammography. The disparity was particularly noticeable in Belgium, where access to all analysed screening tests was lower among non-EU migrants than EU migrants.

The presence of disability did not have a significant influence and was later removed in the final models. The level of education (using lower education as a reference) appeared, conversely, to be a significant factor: the risk of not reporting a mammography was reduced by 30% for persons with the highest educational level, as well as that of not reporting Pap smear testing. The risk of not reporting a colorectal cancer screening was the lowest for those with higher education by 10%, while that of not reporting vaccination against flu was reduced by 35% among persons with a high level of education. Finally, gender was also a significant factor both in colorectal cancer screening and for flu vaccination. There was a higher risk of not reporting colorectal cancer screening among women (+11%) and a lower risk of not reporting the flu vaccination among women (−14%).

## Discussion

Access is considered equitable if it does not depend on, e.g., education, income, migrant status or ethnicity. Comparing utilization between different population groups in order to investigate inequity in access requires adequate indicators on the health care needs. To measure directly the access it is essential to know the population needing for care, which is quite difficult to detect. However, when comparing utilization of preventive services, such as population-based screening for breast cancer, the need is simply defined on the basis of age and gender (every woman in a certain age group is considered to be in need). [24]

Early detection during screening programs for breast, cervical, and colorectal cancer is a key factor for better survival in high-risk groups. Migrants were found to have poorer access to PHSs than nationals, this was particularly true for those from non-EU countries. The comparison among the five EU countries included in the study showed similarities, with slightly poorer access in Italy and with the exception of Malta. Malta showed better access for migrants to PHSs both for female screenings (mammography and Pap smear tests) and for flu vaccination when compared to nationals. Even if the evidence is not strong because of the limited sample size, such a phenomenon is true for any type of screening. A possible explanation is the Maltese public health policy targeting the most vulnerable groups first for screening programmes and vaccination, in order to reduce the burden of access to hospital care [25].

Our findings are in accordance with to previous studies: Visser reported that women born in non-western European countries attend breast cancer screening less frequently than women born in the Netherlands, and register a lower detection rate, which explains a passive attitude to low attendance [26]; previous studies from the United Kingdom, the Netherlands and Spain showed that uptake for cervical screening was lower among migrants compared with non-migrants [27, 28]; similarly, Anson [29] reported that access to preventive services, such as vaccination for tetanus, influenza and rubella, as well as screening for early detection of breast and cervical cancer was better among native Belgians compared with immigrants from Morocco and Turkey even after controlling for socio-demographic characteristics.

In Denmark and Sweden, lower participation rate was recorded for migrant women in mammography screening compared with native-born women. Possible reasons for this are the factors influencing the screening behaviour, namely a lack of knowledge, bureaucratic barriers and cultural issues [30, 31].

In countries with a Beveridge-like health system, such as Italy, Spain and Malta, it should be easier for migrants to access PHSs compared with health systems based on an insurance system, such as in Belgium, or with mixed systems, as in Portugal. Likewise, Francovich and colleagues [14] showed that in Italy migrant access to mammography was as low as 20% compared with native Italians, while the uptake of Pap smear tests was reduced by 35%. Women coming from South America recorded better access to mammography and Pap smear tests, while those from Africa recorded the worst access. For both screenings, a longer length of stay in Italy was positively correlated with better access to PHSs. This supports the hypothesis that the process of integration and/or regularization of migrants, as well as specific interventions for the involvement of foreign women, would facilitate their access to PHSs.

The percentages of women invited by national or regional screening programmes calculated in this study are in line with those from other official statistical sources [32]. The proportion of migrant women participating in organized screening programs was lower than that of nationals, especially for breast cancer screening. Overall, the proportion of women participating in the programmes for cervical cancer screening is very low in Belgium and in Malta. Nevertheless, the proportion of women having a Pap smear test is quite high both for nationals and migrants. This illustrates the serious problems of the screening programs in those countries, partially mitigated by opportunistic screening. In all the countries included in this study, the percentage of women invited by cervical cancer screening programs is similar for natives and migrants, with slightly higher numbers among migrants in Italy and Belgium. In those countries, the screening programs are able to reduce the gap in access to cervical cancer prevention for migrant women.

The proportion of interviewed subjects reporting a colorectal cancer screening was very low (1 out of 7). Only in Italy, there was a significantly lower proportion among migrants, in particular among those from countries outside the EU. This appears to be due to a general lack of knowledge about colorectal cancer, which was more pronounced among some minority groups [33]. Cultural factors may also play a role, e g. fatalism (the perception of everything as being ordained by fate) was found to be associated with a lower uptake of colorectal cancer screening [34]. Our study showed a reduced level of vaccination against flu among migrants, particularly in Italy. This difference was more apparent among non- EU citizens and this result is in line with previous findings from Spain [35] and Italy [36].

Access to PHS for migrants’ access is affected differently at different levels of the health system. Several issues are concerned with equity in health care access, such as entitlements, health policies, structure and organization of services, and the attitude of health professionals towards migrants [24].

The legal framework of entitlement in Belgium, Italy, Portugal and Spain is among the most favourable to everyone living on their territories, including documented and undocumented migrants, among the EU countries. Health policies may facilitate the access of migrants to health services by defining entitlements by law, publicizing them to migrants and health care providers, ensuring appropriate implementation measures, and by not conditioning health care to a person’s immigration status [37]. Italy, Spain and Portugal adopted specific national policies aimed at improving migrant health. Italy and Spain focussed policies on sexual and reproductive health care and communicable disease. [38] Portugal introduced measures which included training, education and communication programmes to inform health professionals of the legal rights of migrants, plus the promotion of partnerships to improve the quality of services provided and facilitate change in organizational culture. [39] Malta adopted public health policy targeting the most vulnerable groups in general, first for screening programmes and vaccination. [25] In contrast, in Belgium there is not yet a clear political commitment to tackling either health inequalities or ethnic inequalities in health, although there is a growing interest regarding this issue. [40] Lack of targeted policies may explain the large gap between nationals and non-EU migrants in accessing female screening and colorectal screening in Belgium, as well as the lack of training of health care professionals. Such a gap is high in Italy as well, notwithstanding the presence of targeted policies, both for EU migrants and non-EU migrant. This can be due to enduring problems in the organization of health services, [41] that may affect more vulnerable groups, who have limited means to overcome the barriers created by poor organization of health services.

In our study, we also considered two important risk factors of inequality in the uptake of vaccination and screening, namely disability and education levels. Disability did not seem to play a role when comparing access to PHSs between migrants and nationals, in contrast to education levels, especially for mammography, Pap smear tests and flu vaccination. Knowledge of differences in health, social and economic conditions is essential for providing equal access to preventive care.

This study was limited by the fact that information on length of stay and area of origin was not considered in detail. In particular, length of stay may play a role in view of the evidence that migrants with a shorter length of stay record lower rates of access/use of health care services, including PHSs [14, 42]. The overall results must be cautiously interpreted because migrant populations in different countries differ in many ways, especially on the rules for acquiring citizenship and migrants’ rights with regard to health care access. This means that conclusions drawn from comparing countries may be more tentative than indicative. Finally, the study only included documented migrants: asylum seekers and refugees are not included in HISs and therefore were not considered in the study.

Many factors may contribute to hindering migrants’ access to PHSs, such as length of stay in host country, religion and levels of literacy. However, using the information available in HISs, only some factors can be investigated, in particular education, gender and the presence of disability.

The major strength of the study is its national representativeness and the excellent comparability of the data among countries, ensured by the harmonisation of data sources at EU level.

## Conclusions

The study provided information about the extent of the gap in accessing PHSs between nationals and migrants in five EU countries. Italy presented the highest gap, while Belgium showed higher risks for non-EU migrants. Poor organization of health services, in Italy, and lack of targeted health policies may explain the findings. In general, only a few prevention programmes exclusively target migrant groups [43]. PHSs should be responsive to patient diversity, probably more than other health services. There is a need for diversity-oriented, migrant-sensitive prevention and a need for prevention programs addressing migrants that are large-scale, evidence-based, sustainable and regularly evaluated. Policies oriented to removing impediments to migrants’ access to preventive interventions are crucial, in order to encourage more positive actions for those facing the risk of intersectional discrimination.

## Appendix

**Table 11:** Description of preventive health services provided in the five European countries surveyed

	Organized screening program	Year of implementation of the screening program	Inclusion of the screening as item of the Health Interview Survey
Screening test			
*Mammography*			
Belgium	Yes	2001	Yes
Italy	Yes	1990	Yes
Malta	Yes	2009	Yes
Portugal	Yes	1990	No
Spain	Yes	1990	Yes
*Pap smear test*			
Belgium	Yes (only Flanders)	2013	Yes
Italy	Yes	1996	Yes
Malta	Yes	2016	Yes
Portugal	Yes	1990	No
Spain	Yes	1986	Yes
*Colorectal screening*			
Belgium	Yes	2009	No
Italy	Yes	2005	Yes
Malta	Yes	2013	No
Portugal	Yes	2009	No
Spain	Yes	2000	Yes

The table contains information about the presence of organized screening program, separately for mammography, Pap smear test and colorectal screening; the year of implementation of the screening program and the inclusion of information about the screenings as an item of the Health Interview Survey in the five studied countries

## Abbreviations

EU: European Union
HISs: Health Interview Surveys
ICC: Intraclass Correlation Coefficient
OR: Odds Ratio
PHSs: Preventive Health Services
USA: United States of America
